# Crystal structure of bis­(isonicotinamide-κ*N* ^1^)bis­(thio­cyanato-κ*N*)zinc

**DOI:** 10.1107/S2056989016008963

**Published:** 2016-06-10

**Authors:** Tristan Neumann, Inke Jess, Christian Näther

**Affiliations:** a Institut für Anorganische Chemie, Christian-Albrechts-Universität Kiel, Max-Eyth Strasse 2, D-24118 Kiel, Germany

**Keywords:** crystal structure, discrete complex, zinc thio­cyanate, isonicotinamide, hydrogen bonding

## Abstract

The asymmetric unit of the title complex, [Zn(SCN)_2_(C_6_H_6_N_2_O)_2_], consists of one Zn^2+^ cation located on a twofold rotation axis, as well as of one thio­cyanate anion and one neutral isonicotinamide ligand, both occupying general positions. The Zn^2+^ cation is tetra­hedrally coordinated into a discrete complex by the N atoms of two symmetry-related thio­cyanate anions and by the pyridine N atoms of two isonicotinamide ligands. The complexes are linked by inter­molecular C—H⋯O and N—H⋯O, and weak inter­molecular N—H⋯S hydrogen-bonding inter­actions into a three-dimensional network.

## Abstract 2

The crystal structure consists of discrete tetra­hedral complexes, that are linked by inter­molecular N—H⋯O, C—H⋯O and N—H⋯O hydrogen bonding.

## Chemical context   

The synthesis of magnetic materials is still a major field in coordination chemistry (Liu *et al.*, 2006 ▸). For their construction, paramagnetic cations can be linked by small anionic ligands such as thio­cyanate anions to enable a magnetic exchange between the cations (Palion-Gazda *et al.*, 2015 ▸; Banerjee *et al.*, 2005 ▸). In this context we have reported on a number of coordination polymers with thio­cyanato ligands that show different magnetic phenomena, including a slow relaxation of the magnetization which is indicative of single-chain magnetism (Werner *et al.*, 2014 ▸; 2015*a* ▸,*b* ▸,*c* ▸). In several cases, such phases can only be prepared by thermal decomposition of suitable precursor compounds (Näther *et al.*, 2013 ▸), leading to microcrystalline powders for which a straightforward crystal structure determination is difficult. In order to avoid this scenario, compounds of the same composition based on cadmium or zinc can be prepared in the form of single crystals. In many cases, such zinc and cadmium compounds are isotypic to the paramagnetic analogues, and the structure of the latter can then easily be refined by the Rietveld method (Wöhlert *et al.*, 2013 ▸). It should be mentioned that the structures of cadmium compounds are useful as prototypes for transition metal compounds with octa­hedral coordination spheres, whereas the structures of zinc compounds are useful prototypes for compounds with tetra­hedral coordination spheres for the transition metal. The thermal decomposition of cobalt complexes is an example of the latter. In the course of our systematic investigation in this regard, we became inter­ested in isonicotinamide as a co-ligand to be reacted with Zn(SCN)_2_. The synthesis and crystal structure of the resulting compound, [Zn(NCS)_2_(C_6_H_6_N_2_O)_2_], are reported here.

## Structural commentary   

**Figure 1:**
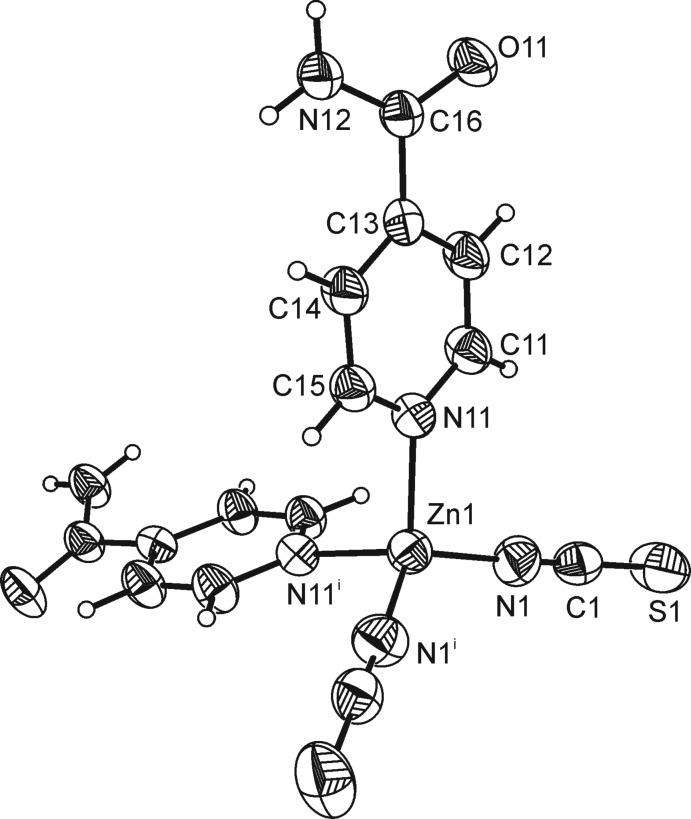
View of the discrete complex with labelling and displacement ellipsoids drawn at the 50% probability level. [Symmetry code: (i) −*x* + 1, −*y* + 1, *z*.]

The asymmetric unit of the title compound consists of one Zn^2+^ cation, one thio­cyanate anion and one neutral isonicotinamide ligand. The thio­cyanate anion and the isonicotinamide ligand are located on general positions whereas the Zn^2+^ cation is located on a twofold rotation axis. The Zn^2+^ cation is tetra­hedrally coordinated by two terminal N-bonded thio­cyanato ligands and by two isonicotinamide ligands through their pyridine N atoms into a discrete complex (Fig. 1 ▸). As expected, the Zn—N bond length involving the thio­cyanate anion (N1) is significantly shorter than that to the pyridine N atom (N11) of the neutral ligand (Table 1 ▸). The angular distortion of the ZnN_4_ tetra­hedron is noticeable, with N—Zn—N angles ranging from 104.32 (13) to 123.6 (2)°.
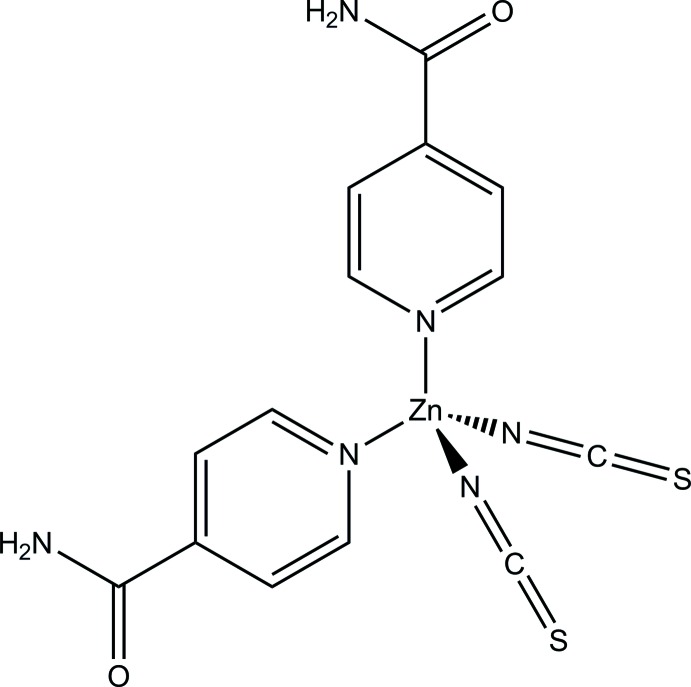


## Supra­molecular features   

**Figure 2:**
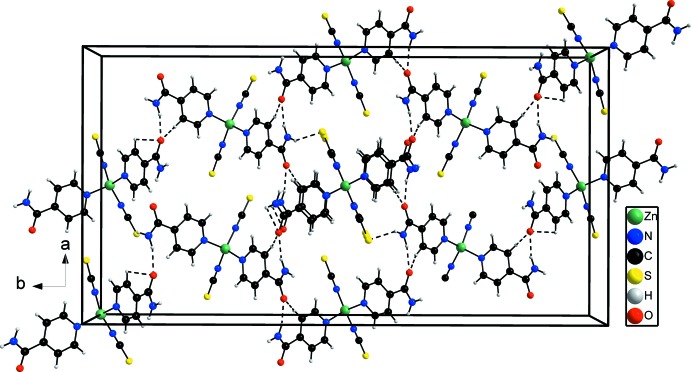
The packing of the complexes in the title compound, in a view along the *c* axis. Inter­molecular hydrogen bonding is shown as dashed lines.

In the crystal structure, the discrete complexes are stacked along the *c* axis and are linked by inter­molecular N—H⋯O hydrogen bonding between one of the two amide H atoms and the amide O atom of a neighboring complex (Fig. 2 ▸ and Table 2 ▸). There is a further weak contact between one aromatic H atom of the pyridine ring and the carbonyl O atom of a neighboring complex (Table 2 ▸). The second H atom of the NH_2_ group is involved in inter­molecular N—H⋯S hydrogen bonding to the S atoms of the anionic ligand. In this way a three-dimensional hydrogen-bonded network is formed.

## Database survey   

To the best of our knowledge, there are only five coordination polymers with isonicotinamide and thio­cyanate anions deposited in the Cambridge Structure Database (Version 5.37, last update 2015; Groom *et al.*, 2016 ▸). This includes two clathrate-structures of Ni compounds with μ-1,3-bridging thio­cyanate anions and with 9,10-anthra­quinone and pyrene as solvate mol­ecules (Sekiya *et al.*, 2009 ▸). Furthermore, a one-dimensional μ-1,3-thio­cyanate-bridged cadmium compound with 9,10-di­chloro­anthracene as clathrate mol­ecule (Sekiya & Nishikiori, 2005 ▸) as well as a three-dimensional network of Cd with μ-1,3-bridging thio­cyanate anions (Yang *et al.*, 2001 ▸) are known. Finally, a compound consisting of Cu^II^–NCS sheets has been reported (Đaković *et al.*, 2010 ▸).

## Synthesis and crystallization   

Ba(NCS)_2_·3H_2_O, ZnSO_4_·H_2_O and isonicotinamide were purchased from Alfa Aesar. Zn(NCS)_2_ was synthesized by stirring 3.076 g Ba(NCS)_2_·3H_2_O (10 mmol) with 1.795 g ZnSO_4_·H_2_O (10 mmol) in 350 ml water. The white residue was filtered off and the filtrate was dried using a rotary evaporator. The homogenity was checked by X-ray powder diffraction and elemental analysis. Crystals of the title compound suitable for single crystal X-Ray diffraction were obtained by the reaction of 27.2 mg Zn(NCS)_2_ (0.15 mmol) with 36.64 mg isonicotinamide (0.3 mmol) in methyl­cyanide (1.5 ml) within a few days.

## Refinement   

Crystal data, data collection and structure refinement details are summarized in Table 3 ▸. C- and N-bound H atoms were located in a difference Fourier map but were positioned with idealized geometry. They were refined with *U*_iso_(H) = 1.2*U*_eq_(C, N) using a riding model with C—H = 0.95 Å for aromatic and N—H = 0.88 Å for the amide H atoms. The absolute structure was determined and is in agreement with the selected setting [Flack *x* parameter: 0.005 (19) by classical fit to all intensities (Flack, 1983 ▸) and −0.005 (8) from 819 selected quotients (Parsons *et al.*, 2013 ▸)].

## Supplementary Material

Crystal structure: contains datablock(s) I. DOI: 10.1107/S2056989016008963/wm5297sup1.cif

Structure factors: contains datablock(s) I. DOI: 10.1107/S2056989016008963/wm5297Isup2.hkl

CCDC reference: 1483379

Additional supporting information: crystallographic information; 3D view; checkCIF report

## Figures and Tables

**Table 1:** Selected bond lengths (Å)

Zn1—N1	1.921 (3)	Zn1—N11	2.033 (3)

**Table 2:** Hydrogen-bond geometry (Å, °)

*D*—H⋯*A*	*D*—H	H⋯*A*	*D*⋯*A*	*D*—H⋯*A*
C14—H14⋯O11^i^	0.95	2.54	3.365 (6)	145
N12—H12*A*⋯S1^ii^	0.88	2.62	3.407 (3)	150
N12—H12*B*⋯O11^i^	0.88	1.97	2.821 (4)	162

Symmetry codes: (i) 
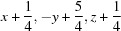
; (ii) 
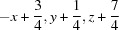
.

**Table 3:** Experimental details

Crystal data	
Chemical formula	[Zn(NCS)_2_(C_6_H_6_N_2_O)_2_]
*M*_r_	425.79
Crystal system, space group	Orthorhombic, *F* *d* *d*2
Temperature (K)	200
*a*, *b*, *c* (Å)	19.1926 (9), 36.3044 (12), 5.2930 (2)
*V* (Å^3^)	3688.0 (3)
*Z*	8
Radiation type	Mo *K*α
μ (mm^−1^)	1.58
Crystal size (mm)	0.20 × 0.16 × 0.11

Data collection	
Diffractometer	Stoe IPDS2
Absorption correction	Numerical (*X-SHAPE* and *X-RED32*; Stoe, 2008 ▸)
*T*_min_, *T*_max_	0.595, 0.742
No. of measured, independent and observed [*I* > 2σ(*I*)] reflections	15338, 2132, 2012
*R*_int_	0.035
(sin θ/λ)_max_ (Å^−1^)	0.662

Refinement	
*R*[*F* ^2^ > 2σ(*F* ^2^)], *wR*(*F* ^2^), *S*	0.031, 0.067, 1.13
No. of reflections	2132
No. of parameters	114
No. of restraints	1
H-atom treatment	H-atom parameters constrained
Δρ_max_, Δρ_min_ (e Å^−3^)	0.24, −0.27
Absolute structure	Flack *x* determined using 819 quotients [(*I* ^+^)−(*I* ^−^)]/[(*I* ^+^)+(*I* ^−^)] (Parsons *et al.*, 2013 ▸).
Absolute structure parameter	−0.005 (8)

Computer programs: *X-AREA* (Stoe, 2008 ▸), *SHELXS97* and *XP* in *SHELXTL* (Sheldrick, 2008 ▸), *SHELXL2014* (Sheldrick, 2015 ▸), *DIAMOND* (Brandenburg, 1999 ▸) and *publCIF* (Westrip, 2010 ▸).

## supplementary crystallographic information

### Crystal data

**Table:**

[Zn(NCS)_2_(C_6_H_6_N_2_O)_2_]	*D*_x_ = 1.534 Mg m^−^^3^
*M**_r_* = 425.79	Mo *K*α radiation, λ = 0.71073 Å
Orthorhombic, *F**d**d*2	Cell parameters from 15683 reflections
*a* = 19.1926 (9) Å	θ = 4.2–56.2°
*b* = 36.3044 (12) Å	µ = 1.58 mm^−^^1^
*c* = 5.2930 (2) Å	*T* = 200 K
*V* = 3688.0 (3) Å^3^	Block, colorless
*Z* = 8	0.20 × 0.16 × 0.11 mm
*F*(000) = 1728	

### Data collection

**Table:**

Stoe IPDS-2 diffractometer	2012 reflections with *I* > 2σ(*I*)
ω scans	*R*_int_ = 0.035
Absorption correction: numerical (*X-SHAPE* and *X-RED32*; Stoe, 2008)	θ_max_ = 28.1°, θ_min_ = 2.2°
*T*_min_ = 0.595, *T*_max_ = 0.742	*h* = −25→25
15338 measured reflections	*k* = −47→47
2132 independent reflections	*l* = −6→6

### Refinement

**Table:**

Refinement on *F*^2^	Hydrogen site location: inferred from neighbouring sites
Least-squares matrix: full	H-atom parameters constrained
*R*[*F*^2^ > 2σ(*F*^2^)] = 0.031	*w* = 1/[σ^2^(*F*_o_^2^) + (0.0282*P*)^2^ + 4.6943*P*] where *P* = (*F*_o_^2^ + 2*F*_c_^2^)/3
*wR*(*F*^2^) = 0.067	(Δ/σ)_max_ < 0.001
*S* = 1.13	Δρ_max_ = 0.24 e Å^−^^3^
2132 reflections	Δρ_min_ = −0.27 e Å^−^^3^
114 parameters	Absolute structure: Flack *x* determined using 819 quotients [(*I*^+^)-(*I*^-^)]/[(*I*^+^)+(*I*^-^)] (Parsons *et al.*, 2013).
1 restraint	Absolute structure parameter: −0.005 (8)

### Special details

**Table:**

Geometry. All esds (except the esd in the dihedral angle between two l.s. planes) are estimated using the full covariance matrix. The cell esds are taken into account individually in the estimation of esds in distances, angles and torsion angles; correlations between esds in cell parameters are only used when they are defined by crystal symmetry. An approximate (isotropic) treatment of cell esds is used for estimating esds involving l.s. planes.

### Fractional atomic coordinates and isotropic or equivalent isotropic displacement parameters (Å^2^)

**Table:**

	*x*	*y*	*z*	*U*_iso_*/*U*_eq_	
Zn1	0.5000	0.5000	0.00134 (10)	0.04197 (15)	
N1	0.41911 (18)	0.48143 (9)	−0.1702 (7)	0.0562 (8)	
C1	0.3688 (2)	0.46990 (10)	−0.2648 (9)	0.0514 (9)	
S1	0.29987 (6)	0.45456 (4)	−0.4003 (3)	0.0802 (4)	
N11	0.46205 (13)	0.53943 (7)	0.2367 (6)	0.0399 (6)	
C11	0.39419 (17)	0.54254 (10)	0.2958 (8)	0.0465 (9)	
H11	0.3616	0.5266	0.2164	0.056*	
C12	0.37026 (17)	0.56804 (10)	0.4673 (8)	0.0466 (8)	
H12	0.3219	0.5697	0.5036	0.056*	
C13	0.41714 (16)	0.59132 (9)	0.5870 (7)	0.0373 (7)	
C14	0.48690 (15)	0.58811 (9)	0.5240 (9)	0.0438 (8)	
H14	0.5205	0.6037	0.6011	0.053*	
C15	0.50720 (17)	0.56217 (10)	0.3489 (7)	0.0428 (8)	
H15	0.5552	0.5604	0.3063	0.051*	
C16	0.39066 (16)	0.61924 (9)	0.7711 (8)	0.0429 (7)	
N12	0.43541 (15)	0.63295 (9)	0.9372 (6)	0.0486 (8)	
H12A	0.4215	0.6495	1.0479	0.058*	
H12B	0.4791	0.6255	0.9367	0.058*	
O11	0.32905 (12)	0.62889 (8)	0.7653 (7)	0.0590 (8)	

### Atomic displacement parameters (Å^2^)

**Table:**

	*U*^11^	*U*^22^	*U*^33^	*U*^12^	*U*^13^	*U*^23^
Zn1	0.0442 (3)	0.0394 (2)	0.0424 (3)	0.0020 (3)	0.000	0.000
N1	0.059 (2)	0.0546 (19)	0.055 (2)	0.0028 (16)	−0.0093 (16)	−0.0096 (15)
C1	0.056 (2)	0.0481 (18)	0.050 (2)	0.0044 (16)	0.0007 (19)	−0.011 (2)
S1	0.0496 (6)	0.0997 (10)	0.0914 (10)	−0.0051 (6)	−0.0034 (6)	−0.0432 (8)
N11	0.0374 (13)	0.0388 (13)	0.0434 (16)	0.0011 (10)	−0.0010 (13)	−0.0002 (13)
C11	0.0353 (16)	0.0455 (17)	0.059 (3)	−0.0030 (14)	−0.0043 (16)	−0.0088 (17)
C12	0.0311 (15)	0.0500 (17)	0.059 (2)	−0.0009 (13)	−0.0041 (15)	−0.0082 (18)
C13	0.0330 (15)	0.0381 (15)	0.0408 (17)	0.0017 (12)	−0.0052 (13)	0.0017 (13)
C14	0.0296 (16)	0.0491 (16)	0.053 (2)	−0.0052 (12)	−0.0021 (17)	−0.0081 (19)
C15	0.0341 (16)	0.0471 (18)	0.047 (2)	−0.0004 (13)	−0.0007 (15)	−0.0036 (15)
C16	0.0327 (14)	0.0492 (17)	0.0467 (19)	0.0021 (12)	−0.0048 (15)	−0.0069 (17)
N12	0.0349 (14)	0.0582 (18)	0.053 (2)	0.0051 (13)	−0.0076 (13)	−0.0151 (15)
O11	0.0322 (12)	0.0719 (17)	0.0728 (19)	0.0104 (11)	−0.0097 (14)	−0.0258 (18)

### Geometric parameters (Å, º)

**Table:**

Zn1—N1^i^	1.921 (3)	C12—H12	0.9500
Zn1—N1	1.921 (3)	C13—C14	1.385 (4)
Zn1—N11	2.033 (3)	C13—C16	1.495 (5)
Zn1—N11^i^	2.033 (3)	C14—C15	1.378 (5)
N1—C1	1.165 (5)	C14—H14	0.9500
C1—S1	1.605 (4)	C15—H15	0.9500
N11—C15	1.336 (4)	C16—O11	1.233 (4)
N11—C11	1.344 (4)	C16—N12	1.326 (5)
C11—C12	1.376 (5)	N12—H12A	0.8800
C11—H11	0.9500	N12—H12B	0.8800
C12—C13	1.387 (5)		
			
N1^i^—Zn1—N1	123.6 (2)	C13—C12—H12	120.2
N1^i^—Zn1—N11	109.39 (13)	C14—C13—C12	117.8 (3)
N1—Zn1—N11	104.32 (13)	C14—C13—C16	122.9 (3)
N1^i^—Zn1—N11^i^	104.32 (13)	C12—C13—C16	119.3 (3)
N1—Zn1—N11^i^	109.40 (13)	C15—C14—C13	119.5 (3)
N11—Zn1—N11^i^	104.42 (17)	C15—C14—H14	120.2
C1—N1—Zn1	177.2 (4)	C13—C14—H14	120.2
N1—C1—S1	178.8 (5)	N11—C15—C14	122.6 (3)
C15—N11—C11	118.2 (3)	N11—C15—H15	118.7
C15—N11—Zn1	118.4 (2)	C14—C15—H15	118.7
C11—N11—Zn1	123.3 (2)	O11—C16—N12	122.1 (4)
N11—C11—C12	122.3 (3)	O11—C16—C13	120.1 (3)
N11—C11—H11	118.9	N12—C16—C13	117.8 (3)
C12—C11—H11	118.9	C16—N12—H12A	120.0
C11—C12—C13	119.7 (3)	C16—N12—H12B	120.0
C11—C12—H12	120.2	H12A—N12—H12B	120.0

Symmetry code: (i) −*x*+1, −*y*+1, *z*.

### Hydrogen-bond geometry (Å, º)

**Table:**

*D*—H···*A*	*D*—H	H···*A*	*D*···*A*	*D*—H···*A*
C14—H14···O11^ii^	0.95	2.54	3.365 (6)	145
N12—H12*A*···S1^iii^	0.88	2.62	3.407 (3)	150
N12—H12*B*···O11^ii^	0.88	1.97	2.821 (4)	162

Symmetry codes: (ii) *x*+1/4, −*y*+5/4, *z*+1/4; (iii) −*x*+3/4, *y*+1/4, *z*+7/4.
